# Long noncoding RNA KCNQ1OT1 promotes colorectal carcinogenesis by enhancing aerobic glycolysis via hexokinase-2

**DOI:** 10.18632/aging.103334

**Published:** 2020-06-21

**Authors:** Cheng Chen, Meng Wei, Chao Wang, Danping Sun, Peng Liu, Xin Zhong, Wenbin Yu

**Affiliations:** 1 Department of General Surgery, Qilu Hospital of Shandong University, Jinan 250012, China

**Keywords:** lncRNA-KCNQ1OT1, HK2, colorectal cancer, aerobic glycolysis

## Abstract

In this study, we investigated the mechanistic role and prognostic significance of the long coding RNA (lncRNA) KCNQ1OT1 in colorectal cancer (CRC). KCNQ1OT1 levels were significantly higher in CRC tissues than adjacent normal colorectal tissues (n=79). High KCNQ1OT1 expression correlated with poorer prognosis in CRC patients. KCNQ1OT1-silenced CRC cells showed reduced proliferation, colony formation, extracellular acidification, and lactate and glucose secretion. This suggests KCNQ1OT1 promotes CRC cell proliferation by increasing aerobic glycolysis. RNA pull-down assays with biotinylated KCNQ1OT1 followed by mass spectrometry analysis showed that KCNQ1OT1 directly binds to hexokinase 2 (HK2). This was confirmed by RNA immunoprecipitation assays using anti-hexokinase 2 antibody. HK2 protein levels were reduced in KCNQ1OT1 knockdown CRC cells, but were restored by treatment with the proteasomal inhibitor MG132. KCNQ1OT1 knockdown CRC cells also showed higher ubiquitinated-HK2 levels, suggesting KCNQ1OT1 enhances aerobic glycolysis by stabilizing HK2. HK2 overexpression in KCNQ1OT1 knockdown CRC cells restored proliferation and aerobic glycolysis. KCNQ1OT1 levels correlated positively with HK2 expression and prognosis in CRC patients. These findings show that KCNQ1OT1 promotes colorectal carcinogenesis by increasing aerobic glycolysis through HK2.

## INTRODUCTION

Colorectal cancer is one of the most malignant tumors worldwide. Despite technological advances in diagnostic and treatment modalities, the prognosis of colorectal cancer patients remains poor because a large number of patients are diagnosed when the cancer has already advanced and therefore are not amenable for surgical resection [1–3]. Furthermore, the precise molecular mechanisms that regulate colorectal carcinogenesis are not well understood [4–7]. Hence, there is an urgent need to identify target genes that can reliably indicate the prognosis of patients with colorectal cancer.

Cancer cells alter their metabolism in order to survive and grow in a highly hypoxic and nutrient-deficient tumor microenvironment [8–10]. It is well documented that cancer cells rewire their metabolism and breakdown glucose to lactate in the presence of oxygen (aerobic glycolysis), a phenomenon called as the Warburg effect [11–13]. This metabolic reprogramming involves altered expression and post-translational modification of several key metabolic enzymes [14]. For example, in human glioblastoma multiforme, the activity of a key glycolytic enzyme phosphoglycerate kinase 1 (PGK1) is enhanced through phosphorylation at Threonine243 [15]. Hexokinases (HKs), which catalyze the first step of glucose metabolism, play a key role in altered metabolism in various tumors [16]. Hexokinase 2 (HK2) is upregulated in head and neck squamous cell carcinoma (HNSCC) and promotes survival of tumor cells by upregulating glycolysis [17–19]. However, the molecular mechanisms underlying the altered expression or post-translational modifications of key metabolic proteins in tumors are yet to be fully understood.

Long non-coding RNAs (lncRNAs) are a class of noncoding RNA molecules that are >200 nucleotides in length [20]. They regulate gene expression at transcriptional and posttranscriptional levels and modulate several cellular functions, including proliferation, differentiation and polarization [21–23]. MIR17HG is an lncRNA that inhibits BLNK protein expression via miR-17-5p, thereby promoting colorectal cancer growth and metastasis [24]. Furthermore, several reports suggest that lncRNAs modulate tumorigenesis by altering glucose metabolism in tumor cells. LncRNA GLCC1 promotes colorectal carcinogenesis and glucose metabolism by stabilizing c-Myc [25]. LncRNA KCNQ1OT1 promotes ovarian cancer progression by upregulating CAPN10 expression through sponging of miR-142-5p [26]. Moreover, lncRNA KCNQ1OT1 promotes osteoblast proliferation, migration, and survival by sponging miR-701-3p and upregulating FGFR3 [27]. However, the role of lncRNA KCNQ1OT1 in tumor glycolysis has not been demonstrated.

In this study, we investigated the mechanistic role and prognostic significance of lncRNA KCNQ1OT1 in colorectal cancer (CRC).

## RESULTS

### KCNQ1OT1 overexpression correlates with poor prognosis in patients with colorectal cancer

**Figure 1:**
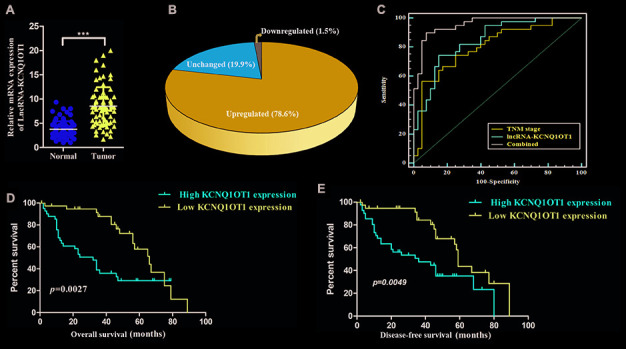
**High KCNQ1OT1 levels correlate with worse prognosis in colorectal cancer patients.** (**A**) QRT-PCR analysis shows that KCNQ1OT1 levels in colorectal tissues compared to adjacent normal colorectal tissue samples from 79 colorectal cancer patients. (**B**) QRT-PCR analysis shows that KCNQ1OT1 levels are 4-fold higher in colorectal tissues compared to adjacent normal colorectal tissue samples from 79 colorectal cancer patients. (**C**) Receiver Operating Characteristic (ROC) curve analysis shows clinical sensitivity and specificity of KCNQ1OT1 expression in 79 colorectal cancer patients. (**D**) Kaplan–Meier survival curve analysis shows overall survival (OS) of high- and low- KCNQ1OT1expressing colorectal cancer patients. (**E**) Kaplan–Meier survival curve analysis shows disease-free survival (DFS) of high- and low-KCNQ1OT1expressing colorectal cancer patients.

Quantitative real time PCR (qRT-PCR) analyses showed that KCNQ1OT1 expression was significantly higher in colorectal cancer samples compared to adjacent normal colorectal tissues (n=79; Figure 1A). As shown in Figure 1B, KCNQ1OT1 levels were at least 4-fold higher in tumor tissues compared to normal colorectal samples. We next analyzed the correlation between KCNQ1OT1 and clinical characteristics of patients with colorectal cancer. The data showed that KCNQ1OT1 expression positively correlated with tumor size and TNM stages (Table 1). Furthermore, receiver operating characteristic curve (ROC) analysis showed that KCNQ1OT1 expression plus TNM stage was a more precise prognostic model than TNM stage alone (Figure 1C). Next, we analyzed the relationship between lncRNA KCNQ1OT1 expression and survival outcomes in colorectal cancer patients. Here, KCNQ1OT1 levels were 4-fold higher in tumor tissues compared with that in the adjacent noncancerous, which were defined as the high expression group. Another was defined as the low expression group. Kaplan-Meier survival curve analysis demonstrated that colorectal cancer patients with high KCNQ1OT1 levels showed significantly lower overall survival (OS) and disease-free survival (DFS) rates than those with low KCNQ1OT1 levels (Figure 1D, 1E). These results demonstrate that KCNQ1OT1 is a potential prognostic biomarker in colorectal cancer.

**Table 1:** Correlation analysis between KCNQ1OT1 levels and clinicopathological features in colorectal cancer patients.

**Characteristics**	**KCNQ1OT1 levels**		***P* value**
	**Low**	**High**	
Age (years)			0.068
<60	27	22	
≥60	10	20	
Gender			0.651
Male	22	22	
Female	15	20	
Tumor size (cm)			0.025
<5	23	15	
≥5	14	27	
Organ location			0.37
Colon	16	23	
Rectum	21	19	
Differentiation			0.819
Well and moderately	14	18	
Poorly	23	24	
Depth of tumor			0.482
T1 +T2	14	13	
T3	12	11	
T4	11	18	
Tumor stage			0.02
I + II	17	10	
III	11	9	
IV	9	23	

### KCNQ1OT1 silencing inhibits colorectal cancer cell proliferation

**Figure 2:**
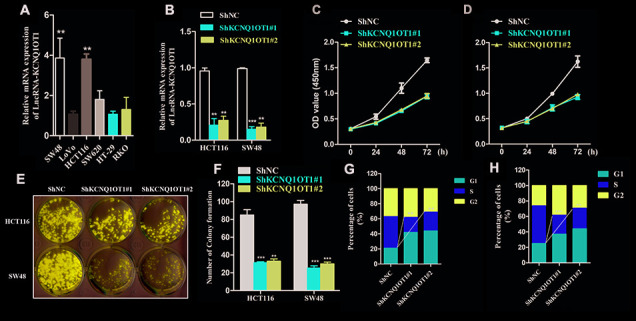
**KCNQ1OT1 silencing inhibits proliferation of colorectal cancer cells.** (**A**) QRT-PCR analysis shows KCNQ1OT1 levels in SW48, LoVo, HCT116, SW620, HT-29 and RKO colorectal cancer cell lines. (**B**) QRT-PCR analysis shows KCNQ1OT1 levels in sh-NC- and sh-KCNQ1OT1-transfected HCT 116 and SW48 CRC cell lines. (**C**, **D**) CCK8 assay results show proliferation status of sh-NC- and sh-KCNQ1OT1-transfected HCT 116 and SW48 CRC cell lines. (**E**) Representative images show colony formation assay results in sh-NC- and sh-KCNQ1OT1-transfected HCT 116 and SW48 CRC cell lines. (**F**) Histogram plot shows total number of colonies in sh-NC- and sh- KCNQ1OT1-transfected HCT 116 and SW48 CRC cell lines. (**G**, **H**) Cell cycle analysis results of Sh-NC- and Sh- KCNQ1OT1-transfected HCT 116 and SW48 CRC cell lines is shown through the flow cytometry analysis. Note: ** denotes p < 0.01 and *** denotes p < 0.001.

Next, we analyzed KCNQ1OT1 levels in several colorectal cancer cell lines. QRT-PCR analysis showed significantly higher levels of KCNQ1OT1 in HCT116 and SW48 cell lines compared to other colorectal cancer cell lines (Figure 2A). Hence, we selected HCT116 and SW48 cell lines for further experiments. We infected HCT116 and SW48 cells with lentiviruses carrying vectors with sh-NC and sh-KCNQ1OT1 constructs and generated stable control and KCNQ1OT1 knockdown cell lines. QRT-PCR analysis showed that KCNQ1OT1 levels were significantly reduced in KCNQ1OT1 knockdown CRC cell lines compared to controls (Figure 2B). CCK-8 assay results showed that proliferation of KCNQ1OT1-silenced CRC cells was significantly reduced compared to the controls (Figure 2C, 2D). Colony formation assay results showed that the total number of colonies were significantly lower in the KCNQ1OT1-silenced CRC groups compared to the controls (Figure 2E, 2F). Cell cycle analysis showed significantly reduced number of S-phase cells in the KCNQ1OT1-silenced CRC group compared to the controls (Figure 2G, 2H). These data demonstrate that KCNQ1OT1is required for the growth and proliferation of colorectal cancer cells.

### KCNQ1OT1 knockdown inhibits aerobic glycolysis in colorectal cancer cells

**Figure 3:**
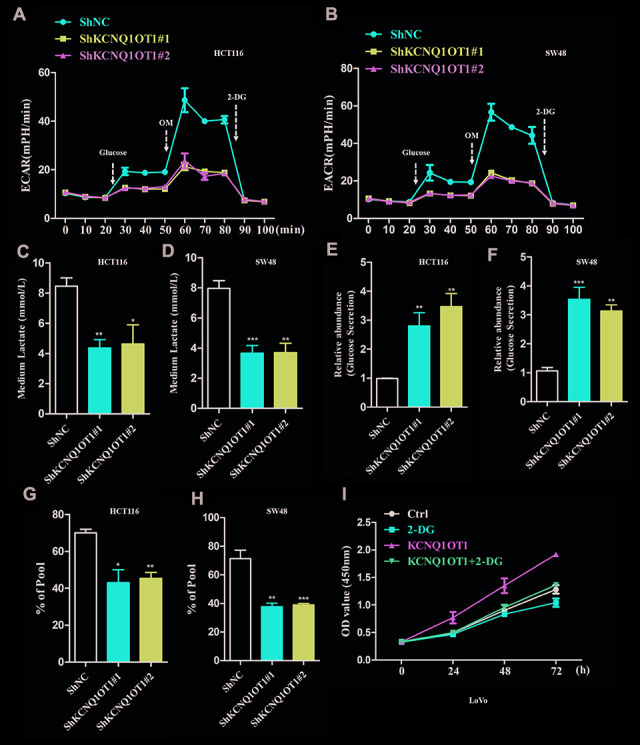
**KCNQ1OT1 silencing inhibits aerobic glycolysis in colorectal cancer cells.** (**A**, **B**) ECAR assay results show extracellular acidification rate of sh-NC- and sh-KCNQ1OT1-transfected HCT116 and SW48 CRC cell lines. Each data point represents means ± SD. (**C**, **D**) Lactate assay results show the levels of lactate in the media of sh-NC- and sh-KCNQ1OT1-transfected HCT116 and SW48 CRC cell lines. (**E**, **F**) Glucose assay results show the glucose levels in the media of sh-NC- and sh-KCNQ1OT1-transfected HCT116 and SW48 CRC cell lines. (**G**, **H**) Metabolic labeling assay results show the ratio of 13C-glucose vs. unlabeled glucose in sh-NC- and sh-KCNQ1OT1-transfected HCT116 and SW48 CRC cell lines. (**I**) CCK-8 assay results show the proliferation status of 2-deoxyglucose-treated control and KCNQ1OT1-overexpressing LoVo cells. Note: *p < 0.05, **p < 0.01, ***p < 0.001.

Next, we analyzed whether KCNQ1OT1 regulates aerobic glycolysis in CRC cells. Extracellular acidification rate (ECAR) assay results demonstrated that extracellular acidification was significantly reduced in KCNQ1OT1-knockdown CRC cells compared to the controls (Figure 3A, 3B). Furthermore, lactate levels were significantly reduced in the media of KCNQ1OT1-knockdown CRC cells compared to the media of the controls (Figure 3C, 3D). Moreover, glucose levels were significantly higher in the media of KCNQ1OT1-knockdown CRC cells compared to the controls (Figure 3E, 3F). ^13^C metabolic flux analysis showed significant reduction ^13^C-labeled metabolites in the media of KCNQ1OT1-knockdown CRC cells compared to the media of the controls (Figure 3G, 3H). Next, we used a glycolytic inhibitor, 2-deoxyglucose (2-DG), to assess whether KCNQ1OT1 affects CRC cell proliferation by regulating aerobic glycolysis. CCK-8 assays showed that proliferation of KCNQ1OT1-knockdown CRC cells was suppressed by treatment with 2-DG (Figure 3I). These data suggest that KCNQ1OT1 promotes CRC cell proliferation and growth by enhancing aerobic glycolysis.

### KCNQ1OT1 directly interacts and stabilizes hexokinase 2 in CRC cells

**Figure 4:**
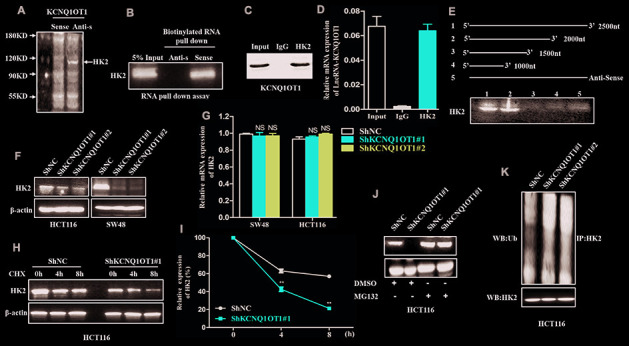
**LncRNA-KCNQ1OT1 directly binds and stabilizes HK2.** (**A**) Proteins retracted from the KCNQ1OT1 pull-down assay are dissected by SDS-PAGE for mass spectrometry assay. (**B**) Western blotting analysis of KCNQ1OT1-interacting proteins that are pulled down in the RNA pull-down assays using biotinylated KCNQ1OT1. (**C**) RNA immunoprecipitation (RIP) assay results show that the KCNQ1OT1 RNA is pulled down with the anti-HK2 antibody in HCT116 cell. (**D**) QRT-PCR analysis of KCNQ1OT1 in the total RNA that is pulled down using the anti-HK2 antibody in the RIP assay, thereby confirming the direct interaction between HK2 protein and KCNQ1OT1. (**E**) Representative western blot images show HK2 protein levels obtained from incubating total protein extracts from HCT116 cells with biotinylated RNAs containing different regions of KCNQ1OT1 and the negative control RNA followed by the RNA pull down assay. The blots are probed using the anti-HK2 antibody. (**F**) Representative western blot images show HK2 protein levels in sh-NC- and sh-KCNQ1OT1-transfected HCT116 cells. (**G**) QRT-PCR results show HK2 mRNA levels in sh-NC- and sh-KCNQ1OT1-transfected HCT116 cells. (**H**) Representative western blot assay results show HK2 protein levels in sh-NC- and sh-KCNQ1OT1-transfected HCT116 cells treated with 100 μg/mL cycloheximide for 0, 4, and 5 h before harvesting the cells for analysis. Untreated cells are used as controls. (**I**) Histogram plot shows HK2 protein levels in sh-NC- and sh-KCNQ1OT1-transfected HCT116 cells treated with 100 μg/mL cycloheximide for 0, 4, and 8 h. (**J**) Representative western blot images show HK2 protein levels in sh-NC- and sh-KCNQ1OT1-transfected HCT116 cells treated with 10mM MG132 for 4h. (**K**) Representative western blot images shows ubiquitinated HK2 protein levels in sh-NC- and sh-KCNQ1OT1-transfected HCT116 cells. Note: **p < 0.01; NS, not significant

We performed a RNA pull down assay using KCNQ1OT1 as bait to identify KCNQ1OT1-interacting proteins. The KCNQ1OT1-binding proteins were separated by SDS-PAGE electrophoresis and analyzed using mass spectrometry and western blotting. Mass spectrometry (MS) and western blotting analyses showed that hexokinase 2 (HK2), a key glycolytic protein, was one of the proteins that binds to KCNQ1OT1 (Figure 4A, 4B). Moreover, RNA immunoprecipitation (RIP) assays using anti-HK2 antibodies pulled down KCNQ1OT1 as analyzed by qRT-PCR analysis of the RNA pulled down by the antibody (Figure 4C, 4D). This suggests that KCNQ1OT1 directly binds to HK2. We further performed RNA pull down assays using different fragments of KCNQ1OT1 as baits to identify the sequence of KCNQ1OT1 that interacts with HK2. RNA pull-down assay followed by western blotting showed that the KCNQ1OT1 nucleotide sequence 1500-2000 is required for HK2 binding (Figure 4E).

Next, we analyzed HK2 mRNA and protein levels in KCNQ1OT1-knockdown CRC cells. The HK2 protein levels were significantly reduced in KCNQ1OT1-knockdown CRC cells compared to the controls (Figure 4F). However, HK2 mRNA levels were similar in control and KCNQ1OT1-knockdown CRC cells (Figure 4G). These data suggest that KCNQ1OT1 regulates HK2 protein expression at the post-transcriptional level. We further treated control and KCNQ1OT1-knockdown CRC cells with a protein synthesis inhibitor, cycloheximide (CHX) to assess the stability of HK2. The results showed that HK2 degradation increased significantly in the KCNQ1OT1-knockdown CRC cells compared to the controls (Figure 4H, 4I). This suggests that KCNQ1OT1 regulates HK2 protein degradation through the ubiquitin-proteasome pathway. The control and KCNQ1OT1-knockdown CRC cells treated with the proteasome inhibitor, MG132, showed similar HK2 protein levels as analyzed by western blotting (Figure 4J). Furthermore, we examined the status of HK2 ubiquitination in control and KCNQ1OT1-knockdown CRC cells. Western blot results showed that the levels of ubiquitinated HK2 protein were significantly higher in the KCNQ1OT1-knockdown CRC cells compared to the controls (Figure 4K). Collectively, these data suggest that direct interaction between KCNQ1OT1 and HK2 inhibits ubiquitination of HK2 and its subsequent degradation through the proteosomal pathway, thereby enhancing its stability.

### KCNQ1OT1 regulates aerobic glycolysis and proliferation via HK2 in CRC cells

**Figure 5:**
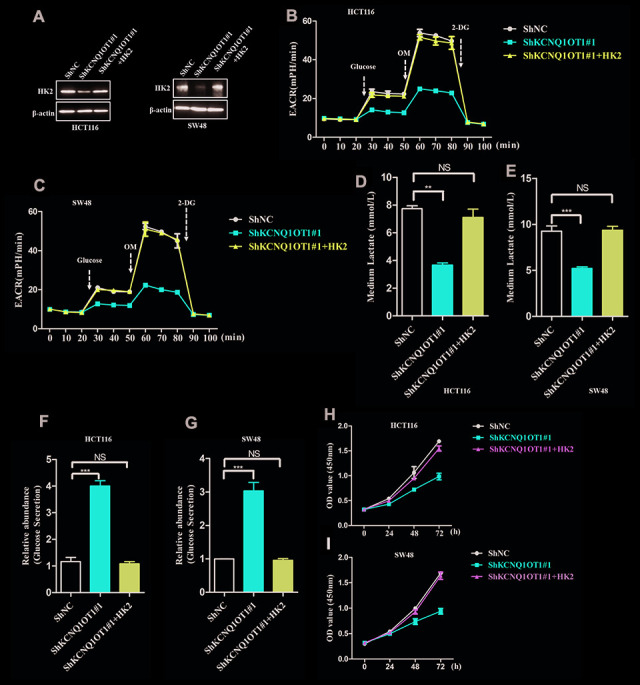
**LncRNA KCNQ1OT1 promotes aerobic glycolysis and CRC cell proliferation via HK2.** (**A**) Representative western blot images show HK2 protein levels in parental HCT116 cells (control) and HK2-overexpressing plus KCNQ1OT1-knockdown HCT116 cells. (**B**, **C**) ECAR assay results show extracellular acidification rate (ECAR) in parental HCT116 cells and HK2-overexpressing plus KCNQ1OT1-knockdown HCT116 cells. Each data point represents means ± SD. The experiment was repeated three times independently. (**D**, **E**) Lactate assay results show lactate levels in parental HCT116 cells and HK2-overexpressing plus KCNQ1OT1-knockdown HCT116 cells. (**F**, **G**) Glucose secretion assay results show the concentration of glucose in the media of parental HCT116 cells and HK2-overexpressing plus KCNQ1OT1-knockdown HCT116 cells. (**H**, **I**) CCK-8 assay results show proliferation status of parental HCT116 cells and HK2-overexpressing plus KCNQ1OT1-knockdown HCT116 cells. Note: **p < 0.01, ***p < 0.001, NS, not significant.

Next, to analyze if KCNQ1OT1 functions as an oncogenic lncRNA in colorectal cancer by regulating HK2 expression, we transfected KCNQ1OT1-knockdown HCT116 cells with the pcDNA3.1-HK2 plasmid and obtained HK2 levels that were similar to parental HCT116 cells as analyzed by western blotting (Figure 5A). HK2-overexpressing KCNQ1OT1-knockdown HCT116 cells showed increased ECAR levels compared to the corresponding controls (Figure 5B, 5C). Furthermore, HK2-overexpressing KCNQ1OT1-knockdown HCT116 cells showed significantly higher levels of lactate in the media compared to KCNQ1OT1-knockdown HCT116 cells, and were similar to parental HCT116 cells (Figure 5D, 5E). Moreover, glucose levels in the media of HK2-overexpressing KCNQ1OT1-knockdown CRC cells were similar to the media of parental HCT116 cells and significantly higher than in the media of KCNQ1OT1-knockdown CRC cells (Figure 5F, 5G). CCK8 assays results showed that proliferation of HK2-overexpressing KCNQ1OT1-knockdown CRC cells was significantly higher compared to the KCNQ1OT1-knockdown CRC cells (Figure 5H, 5I). Taken together, these findings demonstrate that KCNQ1OT1 modulates colorectal cancer proliferation by enhancing aerobic glycolysis through HK2.

### HK2 levels correlate with KCNQ1OT1 expression and prognosis in CRC patients

**Figure 6:**
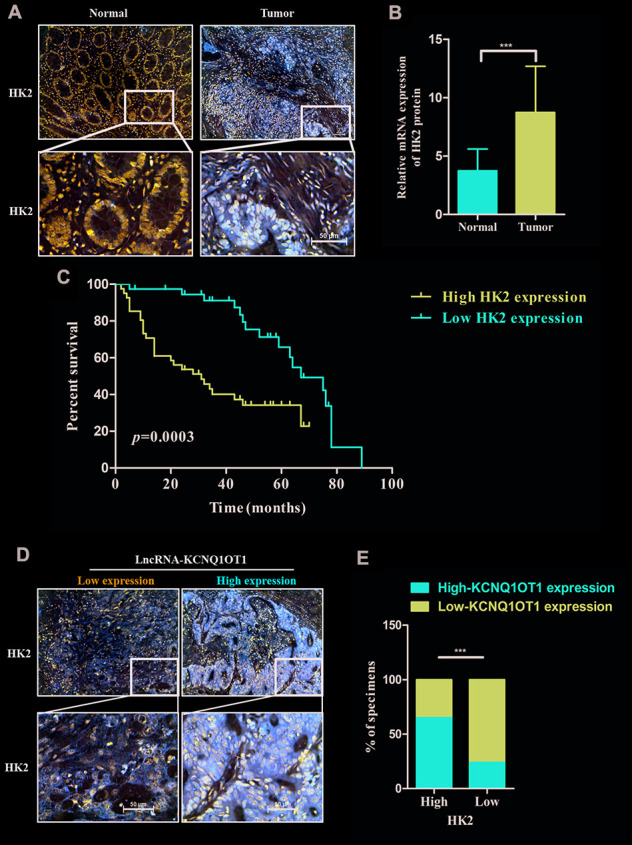
**HK2 protein levels correlate with prognosis and KCNQ1OT1 expression in CRC patients.** (**A**) Representative images show immunohistochemical staining of HK2 protein in 79 pairs of colorectal cancer and adjacent normal colorectal tissues using anti-HK2 antibodies. (**B**) QRT-PCR analysis shows HK2 mRNA levels in 79 pairs of colorectal cancer and adjacent normal colorectal tissues. (**C**) Kaplan–Meier survival curve analysis shows overall survival (OS) of colorectal cancer patients with low and high levels of HK2 mRNA. (**D**, **E**) Correlation analysis shows the relationship between HK2 expression (based on IHC analysis) and lncRNA KCNQ1OT1 levels (qRT-PCR) in 79 colorectal cancer patients. Note: ***p < 0.001.

We next examined HK2 levels in colorectal tumor and adjacent normal colorectal tissues in 79 CRC patients. Immunohistochemical staining results show that HK2 expression is significantly higher in CRC tissues compared to the adjacent normal colorectal tissues (Figure 6A). QRT-PCR results also show that HK2 mRNA levels are significantly higher in CRC samples compared to normal tissues (Figure 6B). Kaplan Meier survival curve analysis shows worse prognosis for CRC patients with high HK2 expression compared to those with low HK2 expression (Figure 6C). Furthermore, KCNQ1OT1 levels (qRT-PCR) positively correlate with HK2 protein expression (IHC data) in CRC patients (Figure 6D, 6E). In conclusion, our data shows that KCNQ1OT1 regulates colorectal carcinogenesis by upregualting aerobic glycolysis via HK2.

## DISCUSSION

Altered metabolism including elevated glycolysis is observed in cancer cells to support enhanced growth and survival in a hypoxic, nutrient-deficient tumor environment [28–30]. Hence, understanding the mechanisms that regulate aerobic glycolysis through abnormal activation of oncogenes or inactivation of tumor suppressors in cancer cells is of paramount importance for developing effective new therapeutic strategies.

Several studies have demonstrated that altered signaling pathways regulate tumor cell growth and metabolism. In breast cancer cells, the transcription factor SIX1 upregulates aerobic glycolysis and tumor growth by increasing the expression of several glycolytic genes [31]. High USP6NL levels in breast cancer cells promote sustained activation of AKT and GLUT1 stability, thereby enhancing aerobic glycolysis [32]. In colorectal cancer cells, elevated caveolin-1 increases glucose uptake and glycolysis [33]. Noncoding RNAs (ncRNAs) also have been reported to regulate tumor progression and aerobic glycolysis by modulating gene expression of metabolic genes at transcriptional and post-transcriptional levels [21]. In hepatocellular carcinoma, miR-139-5p inhibits the expression of glycolytic genes, hexokinase 1 (HK1) and 6-phosphofructo-2-kinase/fructose-2,6-biphosphatase 3 (PFKFB3) expression by directly targeting the ETS1 transcription factor [34]. In colorectal cancer, lncRNA LINRIS increases aerobic glycolysis by stabilizing IGF2BP2 protein and blocking K139 ubiquitination of IGF2BP2 [35]. Our study demonstrates that lncRNA KCNQ1OT1 is significantly upregulated in colorectal cancer tissues compared to adjacent normal colorectal tissues. Moreover, KCNQ1OT1expression inversely correlates with the prognosis of CRC patients. This suggests that KCNQ1OT1 plays a critical role in colorectal carcinogenesis.

Our study shows that KCNQ1OT1 promotes CRC cell proliferation by increasing aerobic glycolysis. In order to proliferate faster, cancer cells rely on the Warburg effect, which involves metabolic reprogramming to increase glucose uptake and enhanced aerobic glycolysis to generate ATP to meet their accelerated growth [36]. KCNQ1OT1 knockdown decreases aerobic glycolysis in colorectal cancer cells. This suggests that KCNQ1OT1 regulates CRC cell proliferation by upregulating aerobic glycolysis. RNA pull-down assay coupled with mass spectrometry analyses demonstrated that KCNQ1OT1 directly interacts with a key glycolytic enzyme, HK2. We demonstrate that the KCNQ1OT1-HK2 interaction inhibits ubiquitination and degradation of HK2. Several reports show that KCNQ1OT1 is distributed in both the nucleus and the cytoplasm [38]. In colon cancer cells, KCNQ1OT1 is mainly distributed in the cytoplasm [39], which is consistent with the findings of our study. We further demonstrate that KCNQ1OT1 levels positively correlate with HK2 expression and prognosis in colorectal cancer patients.

In conclusion, our study demonstrates that high KCNQ1OT1 expression promotes colorectal carcinogenesis by enhancing aerobic glycolysis through direct binding and stabilization of hexokinase 2 (HK2). Hence, KCNQ1OT1 is a potential prognostic indicator and a therapeutic target in colorectal cancer.

## MATERIALS AND METHODS

### Colorectal cancer cell lines

We obtained SW48, LoVo, HCT116, SW620, HT-29 and RKO cell lines from ATCC (Virginia, USA). The cell lines were cultured in high glucose Dulbecco’s Modified Eagle Medium (DMEM; Gibco, USA), supplemented with 10% fetal serum (Gibco, USA), 100 U/mL penicillin and 100 U/mL streptomycin.

### Cell transfections

We purchased lentiviruses containing sh-NC (control) and sh-KCNQ1OT1 from GeneChem (Montreal, Canada). We seeded the HCT116 and SW48 colorectal cancer cell lines in 6-well plates for 24 h and infected with the lentiviruses for another 24 h. Then, the transfected cells were cultured in DMEM medium containing 2 μg/mL puromycin for 72 h. The stably transfected cells were selected by culturing in DMEM medium containing 1μg/mL puromycin for two weeks. We also transfected control pcDNA3.1 (+) plasmid vector and pcDNA3.1 (+)-lncRNA-KCNQ1OT1 into CRC cell lines using lipofectamine 2000 (Invitrogen, CA, USA). Stably transfected cells were selected by growing in DMEM medium containing G418 (400μg/mL, Sigma, USA) for two weeks. We amplified the HK2 gene by using the following PCR primers: forward primer, 5’-ATGATTGCCTCGCATCTGCTT-3’; reverse primer, 5’-CTATCGCTGTCCAGCCTCACG-3’. Then the PCR product was cloned into the pcDNA3.1 (+) vector used PCR SuperMix (TransGen Biotech, Beijing, China) as described by manufacturer’s instructions. Then, we transfected pcDNA3.1 (+)-HK2 and pcDNA3.1 (+)-empty vector into LoVo cells using lipofectamine 2000 (Invitrogen, CA, USA) according to manufacturer’s instructions. Stably transfected cells were selected by growing in DMEM medium containing G418 (400μg/mL, Sigma, USA)) for two weeks.

### Immunohistochemistry (IHC)

We obtained tumor and adjacent normal tissues from 79 colorectal cancer patients that underwent surgical operation at the Shandong Qilu Hospital. The research protocol was approved by the ethics committee of Shandong University (Shandong, China). We obtained signed written consent from all patients involved in this study. For the IHC assay, we incubated 4mm thick sections of paraffin-embedded colorectal tumor and adjacent normal colorectal tissue samples with anti-HK2 antibody (CST, 1:100) at 4°C overnight. Then, the sections were developed using the VisionTMIII Detection System/Mo&Rb (Gene Tech, Shanghai, China) according to manufacturer’s instructions.

### CCK-8 and colony formation assays

For the CCK-8 assay, we seeded 3 × 10^3^ CRC cells from different experimental groups in each well in a 96-well plate. After 24 hours, we added 10 μl CCK-8 (MedChemExpress, Shanghai, China) to all the wells and incubated further for 1.5 h. Then, the absorbance was determined at 450 nm in Multiskan Sky Microplate Reader (Thermo Scientific, MA, USA). For the colony formation assay, 100 CRC cells from different experimental groups were seeded in 6-well plates and cultured for two weeks. The growth medium was changed every three days. Finally, the colonies were stained with crystal violet. We photographed the colonies under the scanner (Canon, China) and counted the total number of colonies in each experimental group.

### RNA Immunoprecipitation (RIP) assay

RIP assays were performed using the Magna RIP™ RNA-Binding Protein Immunoprecipitation Kit (Millipore, USA) according to manufacturer’s protocol. Briefly, 1 × 10^8^ CRC cells were harvested, washed with PBS twice and pelleted down by centrifugation with 1200 g for10 min. Then, the cell pellets were lysed in RIP lysis buffer for 1h. After removing the cell debris, the cell extracts were co-immunoprecipitated with 50 μg/mL rabbit anti-HK2 antibody (CST, USA) or normal mouse IgG in 4°C for 2 h. The unbound material was washed off and the RNA bound to the HK2 protein in the co-IP was isolated with nuclease free water. Then, we performed qRT-PCR analysis using KCNQ1OT1-specific primers to quantify its levels in the retrieved and total cellular RNA.

### RNA pull-down and mass spectrometry (LC-MS) analysis

RNA pull-down assays were performed using the Pierce™ Magnetic RNA-Protein Pull-Down Kit (Thermo Fisher, USA). Briefly, we prepared biotinylated KCNQ1OT1 by *in vitro* transcription using biotin-labeled anti-sense RNA and sense RNA (Invitrogen) according to the manufacturer’s instructions. Then, the biotinylated KCNQ1OT1 RNA was incubated with streptavidin beads and total cell lysates for 2 h. After centrifugation in 4°C for 5 min, we eluted the RNA-protein complexes from the streptavidin beads using 1× SDS loading buffer. The proteins were separated on a 10% SDS-PAGE gels and stained with Coomassie brilliant blue. The protein bands were cut out from the gel and analyzed by mass spectrometry.

### ECAR analysis

ECAR assay was performed using the Seahorse Extracellular Flux Analyzer XF96 (Seahorse Bioscience) according to the manufacturer’s instructions. Briefly, 6 × 10^3^ cancer cells were plated in a XF96-well plate for 24 h. Then, the culture medium was replaced with serum-free DMEM medium for 24 h to starve the cells. Then, unbuffered DMEM medium was added followed by sequential addition of glucose, oligomycin and 2-deoxy glucose (2-DG) up to a final concentration of 10 mM, 1 μM, and 50 mM, respectively. Extracellular acidification rate (ECAR) was measured as mpH/min. ECAR before and after glucose addition is a measure of the glycolytic rate.

### Lactate assay

We seeded 4× 10^3^ CRC cells per well in a 6-well plate for 24 h. Then, we starved the cells with serum-free medium for 24 h and estimated lactate in the media using the L-Lactate Assay Kit (Abcam, CA, USA) according to the manufacturer’s instructions. We normalized the data to total cellular protein concentration in each sample.

### Glucose secretion assay

We seeded 4× 10^3^ CRC cells per well in a six-well plate for 24 h. Then, the medium was removed and replaced with DMEM medium containing 0.1% serum for 24 h. Then, we incubated the cells with glucose-free medium (glucose and phenol red-free DMEM containing 2 mM sodium pyruvate, 20 mM sodium lactate, 2 mM L-glutamine and 15 mM HEPES) for 8 h and estimated glucose concentration in the medium using the Amplex Red Glucose Assay Kit (Thermo Fisher Scientific, USA) according to the manufacturer’s instructions. The data was normalized to total cellular protein concentration in each sample.

### Metabolite analysis of culture media

We seeded 4× 10^3^ CRC cells per well in a six-well plate for 24 h. Then, we replaced the medium with DMEM medium supplemented with 10% dialyzed serum for 24 h. Then, we added ^13^C-labelled or unlabeled glucose (11 mM) into the medium and cultured cells further for another 8h. Finally, we used 200 μl of the cell medium to analyze the labeled metabolites as previously described [37].

### Cell cycle analysis

We harvested the CRC cells, washed twice with ice-cold PBS buffer and then fixed with ice-cold 70% ethanol overnight at -20 °C. The fixed cells were treated with 50 μl of 100 μg/ml RNAse for 15 mins. Then, after centrifugation, the cells were stained with 200 μl of 50 μg/ml propidium iodide for 30 mins in the dark and immediately analyzed by flow cytometry.

### Western blotting

Total protein lysates were prepared by lysing cells with NP40 buffer on ice for 30min and quantified. Then, equal amounts of total protein lysates were boiled with 2× SDS-gel loading buffer and separated on a 10% SDS-PAGE. Then, the separated proteins were transferred onto polyvinylidene difluoride (PVDF) membranes. After blocking the PVDF membranes with 5% skimmed milk for 1 h, they were incubated overnight at 4 °C with primary antibodies against HK2, β-actin, and ubiquitin (all purchased from Cell Signaling Technology, USA). Then, the membranes were incubated for 1 h with the rabbit HRP-conjugated secondary antibody. The membranes were developed using ECL (Boster, China) and the relative amounts of HK2, HK1 and PKM2 were determined using β-actin as loading control. According to the needs of the different experiments, cells were cultured with 10mM MG132 for 4 h or 100 μg/mL cycloheximide for 0, 4, and 8 h before harvested and lysed as performed above.

### Quantitative real-time PCR (qRT-PCR)

We extracted total cellular RNA using TRIzol reagent (Invitrogen, USA). Then, 1 μg of total RNA was reverse transcribed using the PrimeScript RT Reagent Kit (Takara, China). Then, quantitative PCR was performed using the SYBR Green Mix (Takara, China). The amplified transcript level of lncRNA KCNQ1OT1 was normalized to GAPDH using the 2^−ΔΔCt^ method. The primers used in this study were provided below: KCNQ1OT1: F: 5’-GCACTCTGGGTCCTGTTCTC-3’, R: 5’-CACTTCCCTGCCTCCTACAC-3’; GAPDH: F: 5’-CGCTCTCTGCTCCTCCTGTTC-3’, R: 5’-ATCCGTTGACTCCGACCTTCAC-3’.

### Statistical analysis

All data are presented as means ± standard deviation (SD). GraphPad Prism 5 software (GraphPad Software Inc., USA) was used for the statistical analyses. ROC curve was determined by MedCalc statistical software 15.2 (MedCalc Software, Belgium). The cut-off value was calculated as: sensitivity - (1 - specificity). The differences between groups were determined by one-way analysis of variance (ANOVA) or two-tailed unpaired Student's t-test. A value of P < 0.05 was considered significant. All experiments were performed at least three times.
